# PinX1: structure, regulation and its functions in cancer

**DOI:** 10.18632/oncotarget.11411

**Published:** 2016-08-19

**Authors:** Hai-Long Li, Jun Song, Hong-Mei Yong, Ping-Fu Hou, Yan-Su Chen, Wen-Bo Song, Jin Bai, Jun-Nian Zheng

**Affiliations:** ^1^ Jiangsu Key Laboratory of Biological Cancer Therapy, Xuzhou Medical College, Xuzhou, Jiangsu, China; ^2^ Department of Urology, The Affiliated Hospital of Xuzhou Medical College, Xuzhou, Jiangsu, China; ^3^ Jiangsu Center for the Collaboration and Innovation of Cancer Biotherapy, Cancer Institute, Xuzhou Medical College, Xuzhou, Jiangsu, China; ^4^ Department of General Surgery, The Affiliated Hospital of Xuzhou Medical College, Xuzhou, Jiangsu, China; ^5^ Department of Medical Oncology, Huai'an Hospital to Xuzhou Medical College, Huai'an, Jiangsu, China

**Keywords:** PinX1, cancer, structure, regulation, function

## Abstract

PIN2/TRF1-interacting telomerase inhibitor 1 (PinX1) is a novel cloned gene located at human chromosome 8p23, playing a vital role in maintaining telomeres length and chromosome stability. It has been demonstrated to be involved in tumor genesis and progression in most malignancies. However, some researches showed opposing molecular status of PinX1 gene and its expression patterns in several other types of tumors. The pathogenic mechanism of PinX1 expression in human malignancy is not yet clear. Moreover, emerging evidence suggest that PinX1 (especially its TID domain) might be a potential new target cancer treatment. Therefore, PinX1 may be a new potential diagnostic biomarker and therapeutic target for human cancers, and may play different roles in different human cancers. The functions and the mechanisms of PinX1 in various human cancers remain unclear, suggesting the necessity of further extensive works of its role in tumor genesis and progression.

## TELOMERASE AND CANCER

Telomeres are structures composed of tandem repeats of a TTAGGG sequence. They cap the ends of chromosomes, concealing them from the checkpoint responses that recognize broken DNA ends and protecting them from inappropriate exonucleolytic attack and recombination [1]. Maintaining optimal telomere length is crucial for cells. Telomerase is in the core of telomere and composed of protein and RNA components; the major being the catalytic subunit, telomerase reverse transcriptase (TERT) and telomerase RNA component (TERC or TR), which provides an associated RNA template for TERT to codes the telomere repeat. Telomerase can slow or reveres telomere shortening. In most somatic cells, telomerase is insufficient to prevent telomere shortening with cell division and advancing age [2]. Thus, following the division of somatic cells, telomeres will become critically shorten eventually, the ends of chromosomes will lost [3], cells will stop division, replicative senescence occurs [4]. However, telomerase is activated in 90% of all human cancers [5]. In cancer cells TERT is transcriptionally reactivated by the oncogenic transcription factors Myc [6], nuclear factor NF-κB [7], and β-catenin [8]. Thus, oncogene activation leads to telomerase activation, it enables cancer cells become replicative immortality, which is an most important hallmarks of cancer [9]. On the other hand, telomerase in general and its TERT subunit in particular can regulate other various hallmarks of cancer [10], such as regulation of sustaining proliferative signaling [11-14], modulation of angiogenesis [15-19], resistance to apoptosis [20, 21], participation of invasion and metastasis [22-24], maintaining genome stability [25, 26] and so on.

The ability of telomerase to elongate telomeres is regulated by a specialized protein complex, called shelterin or telosome, which binds to and protects telomeres at chromosome ends [27, 28]. In humans, this complex consists of six core proteins: telomeric repeat-binding factor 1 (TRF1 or TERF1) and -2 (TRF2 or TERF2), the single-stranded telomeric DNA binding protein protection of telomeres-1 (POT1), TRF1-interacting nuclear factor 2 (TIN2 or TINF2), TIN2- and POT1-organizing protein (TPP1), and transcriptional repressor/activator protein RAP1 (as known as TERF2IP) [28-30]. This complex is also able to interact with a variety of other proteins by its three telomeric DNA-binding proteins (TRF1, TRF2 and POT1), referred to as the telomere interactome, in order to fulfill their biological functions and control signaling cascades originating from telomeres [31, 32]. TRF1 binds to double-stranded telomeric DNA and plays critical roles in telomere length regulation and telomere protection. It interacts with its associated proteins maintains telomeres at the optimal length [31, 33, 34], PIN2/TRF1-interacting telomerase inhibitor 1 (PinX1) is one of the most important protein in its associated proteins [35]. However, unlike other TRF1-binding proteins, PinX1 is unique in that it can also directly bind to TERT and TERC and inhibit telomerase activity [35, 36].

## REALIZATION TO PINX1

**Figure 1:**
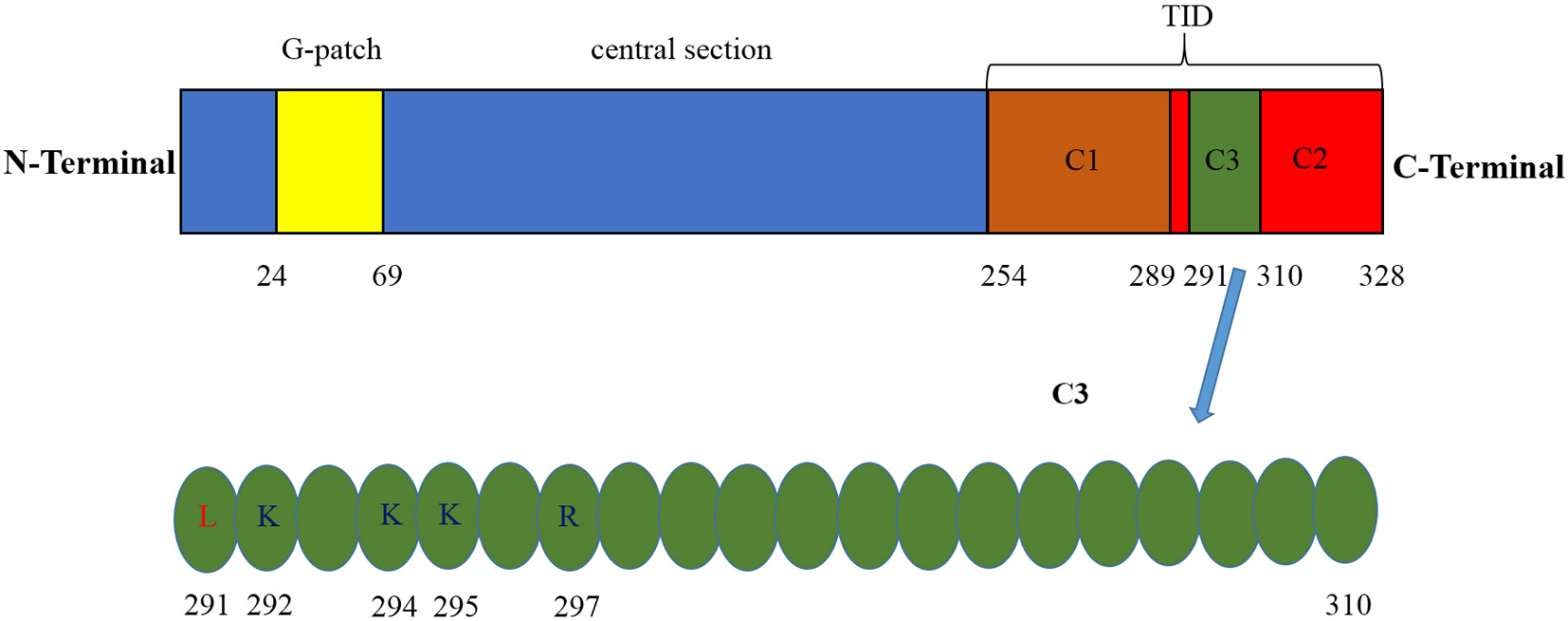
Identification of the main domain for the human PinX1 Top panel, G-patch domain is located at its N-terminal 46aa (24-69aa), TID domain is located at its C-terminal 74 aa (254-328 aa), C1 region of TID domain (254-289aa) specifically binds to Pin2/TRF1, C2 region of TID domain (29-328aa) contains a PinX1 nucleolus localization signal can associate with TERT, C3 region of TID domain (291-310aa) specifically recognized by TRFH domain of TRF1 *via* both hydrophobic as well as hydrophilic interactions. Bottom panel, sequence of C3 fragment. PinX1-L291 is critical for the hydrophobic interaction and PinX1-K292, PinX1-K294, PinX1-K295 and PinX1-R297 are critical for the hydrophilic interaction.

PinX1 is a potent telomerase inhibitor that was first identified and characterized by Zhou et al in 2001 [35]. PinX1 is a 45-kDa protein contains 328 amino acid (aa) in cells. The telomerase inhibitory domain (TID) is located at its C-terminal 74 aa (254-328 aa). The 254-289 aa region in TID domain specifically binds to Pin2/TRF1, this interaction plays a role in the stabilization of telomeres. The 290-328 aa region which contains a PinX1 nucleolus localization signal can associate with TERT, it is essential for the inhibition of telomerase activity [37]. Overexpression of C-terminal TID fragment has stronger depressant effect on telomerase activity in transfected cancer cells [35]. The N-terminal of PinX1 consists of a Gly-rich patch (G-patch) domain. The G-patch domain of PinX1 involves in the process of ribosomal and small nucleolar RNA maturation [38, 39], telomere metabolism [40]. Interestingly, it also has negative influence on PinX1 nucleolar localization [41]. However, it seems that PinX1 without G-patch domain has no effects on telomerase activity than entire PinX1 protein [42]. Thus, the functions of G-patch of PinX1 require thoughtful research. Between the N-terminal and C-terminal is the central section of PinX1, this domain has an effect on mediating TERT nucleolar localization [43] (Figure 1, top panel).

## PINX1 IS A TELOMERASE INHIBITOR ESSENTIAL FOR MAINTAINING TELOMERASE ACTIVITY AND TELOMERE LENGTH

PinX1 can potently inhibit telomerase activation and telomeres elongation in cancer cells [35, 44], this ability is also conserved in yeast, rats and zebra fish [45-47]. Accumulating researches have been executed to dissect the biological function of PinX1 in regulating telomere maintenance. It has been validated that TRFH domain of TRF1 specifically recognizes a 20-amino acid sequence (residues 291-310) of PinX1 by both hydrophobic as well as hydrophilic interactions [48]. PinX1-L291 is critical for the hydrophobic interaction [48, 49] and PinX1-K292, PinX1-K294, PinX1-K295 and PinX1-R297 are critical for the hydrophilic interaction [48] (Figure 1, bottom panel). PinX1 likely interacts with TRF1 in both the nucleolus and the nucleoplasm, forces endogenous TRF1 accumulation in the nucleolus, increases telomere binding of TRF1 [41] and enhances TRF1 stability by inhibiting its degradation and ubiquitination [50]. TRF1-PinX1 interaction is required not only for targeting PinX1 to telomeres but also for PinX1 to prevent telomere elongation in cells. In addition, PinX1 may inhibit telomerase activity by binding to an assembled TERT-TR complex. Thus, this inhibition could occur even at the telomeres, perhaps as a means to fine-tune telomerase-dependent telomere elongation [36].

On the other hand, regulating chromosome stability is another important function of PinX1. Reducing PinX1 by gene knockout not only increases telomerase activity and telomere length, but also leads to chromosome instability in cell models [51]. However, the molecular mechanism by which reducing PinX1 function leads to chromosome instability is not clear. Therefore, further experiments are needed to define how PinX1 controls chromosome stability *via* telomere-dependent and/or -independent telomerase and/or other mechanisms unrelated to telomerase [51].

## PINX1 AND CANCER

PinX1 gene is localized at human chromosome 8p23 [35, 52], which is one of the most frequent Loss of heterozygosity (LOH) regions in human epithelial malignancies, including breast, liver, colon, lung, gastrointestinal and prostate carcinomas et al [53-67]. Moreover, PinX1 overexpression significantly suppresses the growth of hepatocellular carcinoma cells, whereas PinX1 inhibition potently enhances cell growth [52]. Depletion of PinX1 also increases tumorigenicity in nude mice [35]. Thus, PinX1 might be a putative tumor suppressor.

Tumor suppressor genes can be inactivated somatic mutation or deletion or epigenetic inactivation [9]. The mechanism for PinX1 gene inactivation in human cancers is not clear. Akiyama et al [68] examined mutations, mRNA expression and promoter methylation of PinX1 gene in 15 gastrointestinal tract carcinomas (GITC) cell lines, and 20 patients with primary GITC. Only a benign polymorphism had been found. Chang et al [69] assessed for alterations in gene sequence and transcript expression of PinX1, in a series of 52 medulloblastomas, 3 medulloblastoma cell lines and 4 primitive neuroectodermal tumors (PNETs). Direct sequence analysis of all 7 exons and splice junctions of the PinX1 gene revealed no somatic mutations but 11 genetic polymorphisms. Gregory et al [70] performed a detailed DNA sequence analysis of PinX1 in a DNA screening panel of 159 hereditary prostate cancer (HPC) families. They defined 39 polymorphisms and their frequencies in 159 HPC probands. However, PinX1 coding variants seem not be the major factors in increasing the risk for HPC. Oh et al [71] also found two missense mutations of PinX1 in hepatocellular carcinomas (HCCs) and they also revealed no relation with PinX1 expression, telomere length and telomerase activity, suggesting that they are likely polymorphisms. However, PinX1 involved in the telomere length regulation of HCCs indeed. In 2012, Min et al [72] analyzed somatic mutation of PinX1 gene in gastric, colorectal, prostate, breast, and lung carcinomas and concluded that somatic mutational events in the PinX1 may not contribute to development of these carcinomas [72]. Therefore, these combined results suggest that somatic mutation is not the mechanism for inactivation of PinX1. However, LOH of PinX1 loci was found in many carcinomas [53-67]. Thus, LOH seems play a major role in the inactivation of PinX1 in human cancers.

In 2011, Zhou et al [51] investigated that PinX1 is a major tumor suppressor. PinX1 expression is gene dosage-dependent; ablation of one allele reduces protein level by 60-70% *in vitro* and *in vivo* [73], and they generated mice lacking one copy of PinX1, which yielded reduced levels of PinX1 protein, two-fold increased telomerase activity, and an approximately 50% increase in telomere lengths in embryonic fibroblasts (MEFs) and adult tissues [2]. Moreover, PinX1 protein expressions are reduced in human breast cancer tissues and most breast carcinoma cell lines [51]. Furthermore, almost all PinX1^+/−^ mice developed increased rates of cancer, with a shift in tumor spectrum away from the lymphomas and soft tissue sarcomas typical of mice toward epithelial carcinomas more frequently developed in humans, including liver, lung, mammary, and gastrointestinal carcinomas, which are also known to have frequent LOH at 8p23 in humans [51]. Together all of these results indicate that PinX1 is a major tumor suppressor [51]. The mechanisms underlying it may involve telomerase activation and chromosome instability [51].

Assuming a model where the 8p23 region is deleted in one copy of chromosome 8 in a tumor while the remaining copy contains insufficient PinX1 for inhibiting telomerase activity, we may expect that such a tumor will exhibit higher levels of telomerase activity than normal. Ultimately, this telomerase activity increase could transform the tumor into an immortal state caused by telomere lengthening. Some results might confirm this hypothesis. Tomohiro et al [74] revealed that Reduced expression of PinX1 was detected in 68.5% cases of gastric cancer (GC). GC tissues with reduced expression of PinX1 showed significantly higher telomerase activities than those with normal expression of PinX1. LOH of PinX1 locus was detected in 33.3% of 45 cases of GC and was correlated significantly with reduced expression of PinX1. Ma et al [75] reported that the frequency of LOH was higher in GC cases with lymph node metastasis than those without, and was higher in the specimens that were at TNM stage III-IV than those at stage I-II. Thus, PinX1 can be regarded as a sign of gastric cancer development. Then, increasing evidence demonstrate that PinX1 plays a key role in cancer progression. Cai et al [76] performed immunohistochemistry for PinX1 protein on a tissue microarray (TMA) of epithelial ovarian tumors and normal ovaries. They revealed that decreased expression of PinX1 was strongly related to patients with poor prognostic factors regarding presence of lymph node metastasis, distant metastasis, and late International Federation of Gynecology and Obstetrics (FIGO) stage. In univariate survival analysis, a highly significant correlation between loss of PinX1 and shortened patient survival. Liu et al [77] investigated that PinX1 expression in carcinoma of the bladder (UCB) was significantly down-regulated at both mRNA and protein level as compared with that in normal urothelial bladder epithelial tissues. PinX1 levels were inversely correlated with tumor multiplicity, advanced N classification, high proliferation index (Ki-67), and poor survival. Shi et al [78] reported that Reduced expression of PinX1 in prostate cancer (PCa) patients was correlated with advanced clinical stage, high Gleason score, positive regional lymph node metastasis and distant metastasis. Recent years, our research team validated that low PinX1 expression was an independent negative prognostic factor for clear cell renal cell carcinoma (ccRCC), breast cancer (BC) and gliomas patients [79-81]. These results suggest that insufficient PinX1 may involve in the progression of human cancers (Table 1).

**Table 1:** Clinical studies about the relationship between PinX1 and cancer

Published year	Cancer type	Cases of tumor samples	Cases of tumor cell lines	References No.	PinX1 expression
2011	Breast cancer	49	6	51	Decreaesd
2005	Gastric cancer	73	7	74	Decreaesd
2009	Gastric cancer	90	No	75	Decreaesd
2010	Ovarian carcinoma	211	No	76	Decreaesd
2013	Bladder urothelial carcinoma	187	3	77	Decreaesd
2014	Prostate cancer	40	No	78	Decreaesd
2015	Clear cell renal cell carcinoma	278 and 35	2	79	Decreaesd
2015	Breast cancer	405	2	80	Decreaesd
2013	Esophageal squamous cell carcinoma	98 and 59	No	82	Overexpressed
2014	Cervical squamous cell carcinomas	122	5	83	Overexpressed

However, some researches showed opposing molecular status of PinX1 gene and its expression patterns in other types of tumors. Qian et al [82] demonstrated that PinX1 was frequently overexpressed in esophageal squamous cell carcinoma (ESCC) tissues. The expressions of PinX1 were detected by immunohistochemistry performed in two independent cohorts of ESCC patients and by western blotting in five ESCC cell lines. Similarly, Tian et al [83] found the PinX1 expression level is upregulated in primary cervical squamous cell carcinomas (CSCC) tissues as well as in five CSCC cell lines. High expression of PinX1 was also examined over 50% of advanced CSCC patients. These suggest that the abnormalities and/or functions of PinX1 in tumour genesis and progression are complicated and may be tumor-type-specific.

The mechanism of PinX1 function in human cancer cells has yet to be fully elucidated. As we introduced previously, it was originally identified as an telomerase inhibitor and involved in maintaining telomerase activity, telomere length and chromosome stability. Besides its binding to and inhibition of telomerase, other mechanisms of these functions also been found, for example: the regulation of telomerase activity by PinX1 in gastric cancer is involved in Mad/c-Myc pathway [84], and the role of PinX1 in telomerase function regulation is mediated by cycle-dependent localization of telomerase to telomere [85]. Furthermore, PinX1 is a novel microtubule-binding protein essential for faithful chromosome segregation in mitosis. Suppression of PinX1 by siRNA abrogates faithful chromosome segregation and results in anaphase chromatid bridges in mitosis and micronuclei in interphase, suggesting an essential role of PinX1 in chromosome stability [86]. In addition, the promoter region of human PinX1 gene also had been cloned and characterized and several putative binding sites for transcription factors such as CREB, P53, E2F, GATA-1, USF, HNF, NF-κB and C/EBP had been revealed. These reveals could facilitate further studies to explore the regulation and conversion mechanism of PinX1 expression during oncogenesis [87]. Recently, it was been demonstrated that PinX1 expression is directly activated by P53 in cervical cancer cells. HPV16 E6, which plays an important role in human cervical carcinogenesis [88], can inhibit the expression of PinX1 *via* suppressing P53 transcriptional activity, resulting in the enhancement of telomerase activity [89]. Our research also identified that PinX1 inhibited the migration and invasion of ccRCC cells and BC cells by suppressing MMP-2 and MMP-9 expression and activity respectively *via* NF-κB-dependent transcription *in vitro* [79, 80]. It seems further works are needed to clarify the mechanisms of PinX1 in regulating tumor genesis and progression of different types of human carcinomas in detail.

## PINX1 IS A POTENTIAL TARGET IN CANCER THERAPY

As a major tumor suppressor, PinX1 has been demonstrated to be a new potential target in cancer therapy. Zhou et al firstly showed that overexpression of PinX1 (especially its small TID domain) decreased cancer cells' tumorigenicity in mice [35]. In our previous study, we demonstrated that PinX1 suppresses renal cell carcinoma and breast cancer invasion and metastasis *in vitro* and *in vivo* [79, 80]. Subsequently, this result has also been confirmed in other tumor cells just like human fibrosarcoma cells, hepatocellular carcinoma cells, breast cancer cells, burkitt's lymphoma cells, nasopharyngeal carcinoma cells and esophageal cancer cells [51, 90-94]. Moreover, some researchers expanded this application to many different human cancer cells or tissues by various schleppers. Chen et al fused TID domain of PinX1 to the transactivator of transcription of human immunodeficiency virus (Tat) - an 11-amino acid peptide that translocates across the cell membrane. This purified protein was efficiently transduced into the hepatocellular carcinoma cell and hepatoblastoma cell and caused telomere shortening, limited their proliferation and inhibited growth of tumors from these cells in mice [91]. Zhang et al generated novel recombinant proteins containing mBAFF (a novel ligand for targeted therapy of BAFF receptor-positive malignancies), a polyarginine tract 9R and PinX1 (or its C/N terminal), to target lymphoma cells. These fusion proteins specifically killed BAFF receptor-expressing Burkitt's lymphoma (BL) cells by inhibiting telomerase activity. Therapeutic experiments using fusion protein containing C terminal of PinX1 in SCID mice implanted with Raji cells showed significantly prolonged survival times, indicating the *in vivo* antitumor activity of the fusion protein. In 2014, Long et al combined doxorubicin with short interfering siRNA and a novel nanoparticle. This compound was shown to be viable for siRNA deliver into glioma cells and had more effective role in inhibiting gliomas [95]. Moreover, PinX1 overexpression could enhance the sensitivity of human gastric carcinoma cell line to 5-fluorouracil [96]. Interestingly, knockdown of PinX1 dramatically enhanced paclitaxel cytotoxicity to cervical squamous cell carcinomas cells [83] and knockdown of PinX1 substantially increased ESCC (oesophageal squamous cell carcinoma) cells' therapeutic efficacy of radiation both *in vitro* and *in vivo*.

## CONCLUSIONS AND OUTLOOK

PinX1 is identified as a TRF1/Pin2-binding protein and a telomerase inhibitor originally. Then it has been demonstrated that it is essential for maintaining telomeres length and chromosome stability. Recent results have discovered that PinX1 is a major tumor suppressor and involved in tumorigenesis and tumor progression. Furthermore, emerging evidence suggest that PinX1 (especially its TID domain) might be a potential new target cancer treatment. However, many questions remained to be addressed including what are the functions of G-patch of PinX1, how PinX1 regulates tumorigenesis and tumor progression, what induce the functions of PinX1 to be tumor-type-specific, how to develop PinX1-based cancer diagnosis and individualized treatment, and whether PinX1 has other new functions. Further works on this novel protein would reveal novel insight into the regulation of tumor genesis and progression and might eventually lead to new diagnosis and individualized treatment for cancers.
